# *Pinelliae Rhizoma Praeparatum Cum Alumine* Extract: Sedative and Hypnotic Effects in Mice and Component Compounds

**DOI:** 10.1155/2019/6198067

**Published:** 2019-04-28

**Authors:** Sisi Lin, Bo Nie, Ke Song, Ren Ye, Zhengzhong Yuan

**Affiliations:** ^1^Department of Traditional Chinese Medicine, The First Affiliated Hospital of Wenzhou Medical University, Wenzhou 325000, China; ^2^Key Laboratory of Chinese Internal Medicine of Ministry of Education and Beijing, Dongzhimen Hospital, Beijing University of Chinese Medicine, Beijing 100700, China

## Abstract

*Pinelliae Rhizoma Praeparatum Cum Alumine* (PRPCA) is useful for eliminating dampness and phlegm in clinical settings, targeting the main mechanisms of insomnia as defined in traditional Chinese medicine. However, little is known regarding the sedative and hypnotic effects of PRPCA. In the present study, we examined the sedative effects of PRPCA via a locomotor activity test and aimed to determine the most appropriate concentration of PRPCA for achieving these effects. The strongest sedative effects were observed at a PRPCA concentration of 0.45 g/ml. In addition, we investigated the hypnotic effects of PRPCA and its role in promoting sleep via sleep monitoring and vigilance state analysis. PRPCA increased rapid eye movement (REM) sleep and non-REM (NREM) sleep while decreasing wakefulness. In addition, PRPCA decreased the number of bouts of wakefulness (16–32 s and 32–64 s) and increased the number of bouts of NREM sleep (128–256 s). Furthermore, we identified a total of 32 component compounds via chromatography and mass spectrometry. Hence, the current work provides valuable information regarding the sedative and hypnotic effects of PRPCA and its regulatory mechanisms in promoting sleep.

## 1. Introduction

Insomnia is a common and significant public health problem worldwide [1], exerting substantial effects on society and the economy due to workplace absenteeism and accidents and decreases in productivity [2, 3]. The treatment of insomnia can be complex and time-consuming for both patients and providers. Effective treatment requires appropriate diagnosis, cognitive behavioral therapy for insomnia (CBT-I), and pharmacological treatment. Although CBT-I is regarded as a first-line treatment option [4], there are still several barriers that limit its use, including a lack of qualified clinicians, geographical remoteness, stigma associated with receiving psychological services, perceived costs, and the dissemination of inadequate information among patients and providers [5–7]. Considering their side-effects, pharmacological agents should be prescribed with caution and are recommended for short-term use only. Given these limitations, researchers worldwide have begun to focus on developing safer, more effective, and more economical treatment strategies for insomnia.

In traditional Chinese medicine, phlegm stagnation and disconnection between Yin and Yang are the main causes of insomnia.* Pinelliae Rhizoma* (PR) can treat insomnia by eliminating dampness and phlegm and connecting Yin and Yang [8]; it also reduces the incidence of adverse reactions and alleviates vomiting [9, page 119] . The first recorded use of PR for the treatment of insomnia is described in* HuangdiNeijing*, a classic work in traditional Chinese medicine. Song et al. [10] noted that there are 177 prescriptions for insomnia described in the* HuangdiNeijing* and Chinese medical literature published no later than 1911, including 159 prescription drugs. Among them, PR is listed as the sixth most utilized drug. However, considering the toxicity of its raw form, PR is usually processed in order to enhance its efficacy and reduce toxicity. In our recent study [11], we observed that the* Pinellia Ternata (Thunb.)Makino* preparation—which is prepared by processing raw PR with licorice, lime, and alum as adjuvant materials—promotes sleep and sedation in mice by increasing REM sleep. PRPCA is another preparation of PR with a simpler preparation process. In this preparation, the quality of the drug is affected by fewer factors, and PRPCA is relatively easier to obtain with better quality control. PRPCA is processed after soaking PR in* Alumine* solution, which is an astringent that effectively eliminates dampness. This procedure reduces the toxicity of the drug as well as irritation, enhancing its ability to eliminate dampness and phlegm. According to the Pharmacopoeia of the People's Republic of China (2015), PRPCA exhibits lower toxicity than raw PR [9, page 120]. Tao et al. [12] compared the toxicity and irritating effects of raw PR, PR processed with ethanol, and PRPCA and reported that PR processed with ethanol and PRPCA significantly reduced irritation in the rabbit conjunctiva and prostaglandin E2 (PGE2) content (an inflammatory mediator) in peritoneal exudates from rats. Although this study highlighted the safety of PRPCA, little is known regarding its sedative and hypnotic effects.

## 2. Materials and Methods

### 2.1. Experimental Design

The present study included three parts: (a) investigation of the sedative effects of PRPCA in mice and the proper dose of PRPCA; (b) investigation of the hypnotic effects of PRPCA in mice and its role in promoting sleep; (c) identification of the chemical components of PRPCA.

### 2.2. Drugs and Reagents

PRPCA is a type of PR preparation (i.e., dried tuber of* Pinellia Ternate *(*Thunb*.)* Breit.*) that is processed with alumine as an adjuvant material. PRPCA granules (batch number: 1510002S) were purchased from China Resources Sanjiu (Beijing) Pharmaceutical Co., Ltd (Beijing, China), and identified by Xiaochen Chen, the deputy director of the TCM pharmacy at the First Affiliated Hospital of Wenzhou Medical University in Zhejiang, China. Diazepam (DZP, batch number: AH120701) was purchased from Shanghai XudongHaipu Pharmaceutical Co., Ltd. (Shanghai, China) for use as a positive control. Sterile water for injection (SWFI, batch number: M11073107) was purchased from Fuzhou Haiwang Pharmaceutical Co., Ltd. (Fujian, China) for use as a placebo and solvent. High-performance liquid chromatography- (HPLC-) grade acetonitrile was purchased from J.T.Baker Company (J.T.Baker, USA). Ultrapure water produced using a MilliQ water purification system (18.2 MΩ, Millipore, Bedford, MA, USA) was used in all aqueous solutions.

### 2.3. Drug Preparation and Extraction

Each bag of PRPCA contained 0.5 g of PRPCA granules, which is equivalent to 6 g of raw PRPCA. In our preliminary experiment [13], 4.5 bags, 9 bags, and 18 bags of PRPCA granules were dissolved in SWFI and titrated to 60 ml to yield concentrations of 0.45 g/ml, 0.9 g/ml, and 1.8 g/ml (equal to treatment doses of 9 g/kg, 18 g/kg, and 36 g/kg when 0.02 ml/g was intragastrically administered to mice), respectively. The prepared solutions were stored in a refrigerator with a temperature below 4°C. One tablet of DZP was dissolved in 25 ml of SWFI to obtain 0.1 mg/ml of liquid DZP.

PRPCA granules were crushed and soaked overnight in a cold acetonitrile: water solution (3:1, v/v), following which they were subjected to 45 min of ultrasonification for extraction. The extract was centrifuged at 10,000 r/min for 10 min. The supernatant was filtered through a 0.22 *μ*m microporous membrane and stored at −80°C for liquid chromatography–mass spectrometry (LC–MS) analyses.

### 2.4. Animals and Grouping

Male SPF C57BL/6J mice weighing 20–25g (10–12 weeks old) were used for the present study. Mice were supplied by the Laboratory Animal Centre at the Chinese Academy of Sciences (Shanghai, China). The animals were individually housed at a constant temperature (22±0.5°C) and relative humidity of 60%±2%. Animals were housed under a 100-lux 12-hour light/dark cycle (lights on 7:00 AM to 7:00 PM) and were provided* ad libitum* access to food and water. This study was performed in accordance with the recommendations of the National Institutes of Health Guide for the Care and Use of Laboratory Animals and the experimental laboratory animal ethics committee of the Laboratory Animal Center at Whenzhou Medical University. The protocol was approved by the experimental laboratory animal ethics committee of the Laboratory Animal Center at Whenzhou Medical University (wydw 2016-026).

For the locomotor activity test, all 30 mice were randomly divided into the following five groups: (1) the placebo group, in which mice were intragastrically administered SWFI; (2) the positive control group, in which mice were intragastrically administered DZP at 0.1 mg/ml; (3) the PRPCA 1 group, in which mice were intragastrically administered PRPCA at 0.45 g/ml; (4) the PRPCA 2 group, in which mice were intragastrically administered PRPCA at 0.9 g/ml; and (5) the PRPCA 3 group, in which mice were intragastrically administered PRPCA at 1.8 g/ml.

For sleep monitoring and vigilance state analyses, all 18 mice were randomly divided into the following three groups: (1) the placebo group: mice were intragastrically administered SWFI; (2) the positive control group: mice were intragastrically administered DZP at 0.1 mg/ml; and (3) the PRPCA group: mice were intragastrically administered PRPCA at 0.45 g/ml. All drug volumes were calculated based on the weights of the mice, as follows: mouse weight×0.02.

### 2.5. Locomotor Activity Test

To investigate the sedative effects of different concentrations of PRPCA and determine the most effective concentration, we assessed locomotor activity in experimental mice.

Each mouse was housed in an individual recording container (28cm×16.5cm×13cm) with a passive infrared sensor (Biotex, Kyoto, Japan) placed 17.5 cm above the floor of the container, as previously reported [14]. All detectors were connected to the computer to record mouse movement during the test. Prior to behavioral recording, all mice were habituated to the container for 24 h. Then, the drugs were intragastrically administered to mice at 7:00 PM for 14 days. Locomotor activity was continuously recorded during the 14-day period, and the data were fed back to the computer every 5 min. Locomotor activity for each 24 h period was obtained by summing the corresponding records from 7:00 PM on one day to 7:00 PM on the following day.

### 2.6. Sleep Monitoring and Vigilance State Analyses

Under 5% chloral hydrate anesthesia (400 mg/kg, i.p.), mice were simultaneously implanted with electrodes for polysomnographic electroencephalography (EEG) and electromyography (EMG) recording [15]. Briefly, two stainless steel screws (1 mm in diameter) were inserted through the skull above the cortex (anteroposterior, +1.0 mm; left–right, −1.5 mm from bregma or lambda) [16] to monitor EEG signals, and two insulated stainless steel screws were placed bilaterally into both trapezius muscles to function as EMG electrodes. All electrodes were attached to a microconnector and fixed to the skull using dental cement, following which the wounds were closed.

A slip-ring was designed to ensure that the behavioral movement of the mice would not be restricted during EEG and EMG recording [17]. After a 7-day recovery period, mice were individually housed in transparent barrels and habituated to the recording cable for 24 h prior to sleep monitoring. Then, the drugs were intragastrically administered to mice at 7:00 PM for 14 continuous days. After this 14-day period, sleep–wakefulness states were monitored for 24 h.

The EEG/EMG signals were amplified, filtered (EEG: 0.5–30 Hz; EMG: 20–200 Hz), digitized at a sampling rate of 128 Hz, and recorded using SleepSign software (Kissei Comtec, Nagano, Japan), as described elsewhere [18, 19]. Vigilance was then automatically classified offline under 4 s epochs into wakefulness, rapid eye movement (REM) sleep, and non-REM sleep (NREM) using SleepSign software, in accordance with standard criteria [20]. Wakefulness is characterized by low-amplitude, high-frequency EEG activity, and high EMG activity. REM sleep is characterized by low-amplitude, high-frequency EEG activity, and an absence of EMG activity. The presence of EEG theta activity (6–9Hz) can be used to confirm this state. NREM sleep is commonly characterized by high-amplitude EEG activity and low-voltage EMG activity. The presence of high delta activity on EEG (0.65–4 Hz) is also used to define this state. Finally, the defined sleep–wake stages were visually examined and corrected if necessary.

### 2.7. Chromatography and Mass Spectrometry

The samples were analyzed using a Waters Acquity ultra-performance liquid chromatography system (UPLC, Waters Corporation, Milford, MA, USA) equipped with an ACQUITY UPLC HESS C18 column (2.1 mm × 100 mm, 1.8 *μ*m). The UPLC system consisted of a vacuum degasser, a binary pump, an autosampler, a column heater, and a diode array detector (DAD) coupled to a quadrupole time-of-flight mass spectrometry (QTOF) analyzer in a SYNAPT G1 system (Waters Corporation) and equipped with an electrospray ionization (ESI) interface.

Water served as mobile phase A, while acetonitrile served as phase B. The temperature of the column was set to 40°C. The elution program was as follows: 5–95% B from 0 to11.5 min, 95% B from 11.5 to 13.5 min in positive ionization mode, and 5–95% B from 0 to17min/95% B from 17 to 19 min in negative ionization mode. The injection volume was 2 *μ*l, and the flow rate was set at 0.4 ml/min.

Parameters for analysis were set using ESIin both the positive and negative ionization modes (ESI + and ESI–). Ultra-high pure helium (He) was used as the collision gas, while high purity nitrogen (N2) was used as the nebulizing gas. The parameters were set as follows: cone voltage: 40V; capillary voltage for ESI+: 3,000V; capillary voltage for ESI–: 2,500V; cone gas rate: 50 L/h; desolvation gas rate; 800 L/h, source temperature: 100°C; mother ion collision energy: 6 eV. The data acquisition rate was 0.2s. The mass range was scanned from Da 100–1,200.

The raw data acquired via UPLC/Q-TOF-MS were analyzed using Marker Lynx Applications Manager version 4.1 (Waters, Manchester, UK). This allowed for data pretreatment procedures such as peak detection, deconvolution, normalization, alignment, and data reduction to give a list of mass and retention times that paired with the corresponding intensities for all detected peaks from each data file in the dataset.

### 2.8. Statistical Analysis

All data were expressed as the mean ± the standard error of the mean (SEM). Statistical analyses were performed using SPSS 19.0(IBM, New York, USA). Locomotor activity data were assessed using repeated-measures analyses of variance (ANOVA). Sleep monitoring data were analyzed using one-way ANOVA, following which the least significant difference (LSD) method was used for comparisons among groups. In all cases, P values < 0.05 were considered statistically significant.

## 3. Results

### 3.1. Sedative Effects of PRPCA in Mice

**Figure 1:**
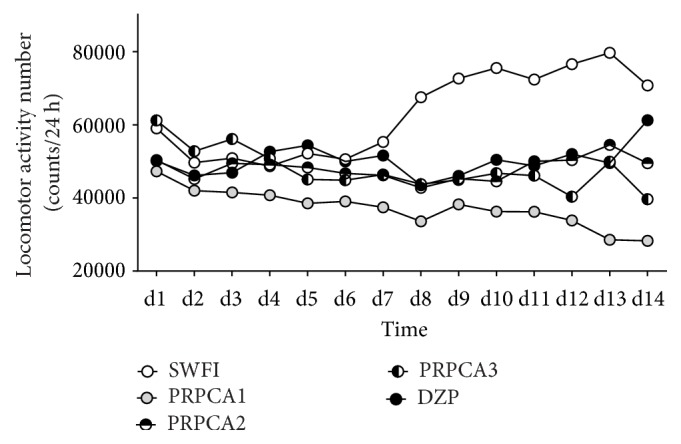
*Locomotor activity following intragastric administration*. White, gray, horizontal black/white, vertical black/white, and black circles represent the profiles for the SWFI, PRPCA 1, PRPCA 2, PRPCA 3, and DZP group, respectively. SWFI: sterile water for injection; PRPCA:* Pinelliae Rhizoma Praeparatum Cum Alumine;* DZP: diazepam.

We evaluated locomotor activity data for each day after intragastric administration to examine the sedative effects of PRPCA in mice and determine the optimal concentration for achieving these effects. The results are shown in Tables 1, 2, and 3 and Figure 1.

Although significant differences in locomotor activity were observed among the groups (F=4.267, P≤0.001) (Table 1), no such differences were observed among the different time points (F=2.133, P=0.088). We also observed an interaction between group and treatment time (F=6.242, P=0.004). At the same time point, we observed significant differences among the groups from days 8 to 14 (Table 2).  Table 3 shows that PRPCA 1, PRPCA 2, PRPCA 3, and DZP reduced the total locomotor activity in mice, when compared with SWFI (P≤0.001, P=0.012, P=0.010, and P=0.027, respectively). Locomotor activity was lower in the PRPCA 1 group than in the remaining four groups from days 1 to 14 (Figure 1).

### 3.2. PRPCA Decreased Wakefulness and Increased Sleep in Mice

**Figure 2:**
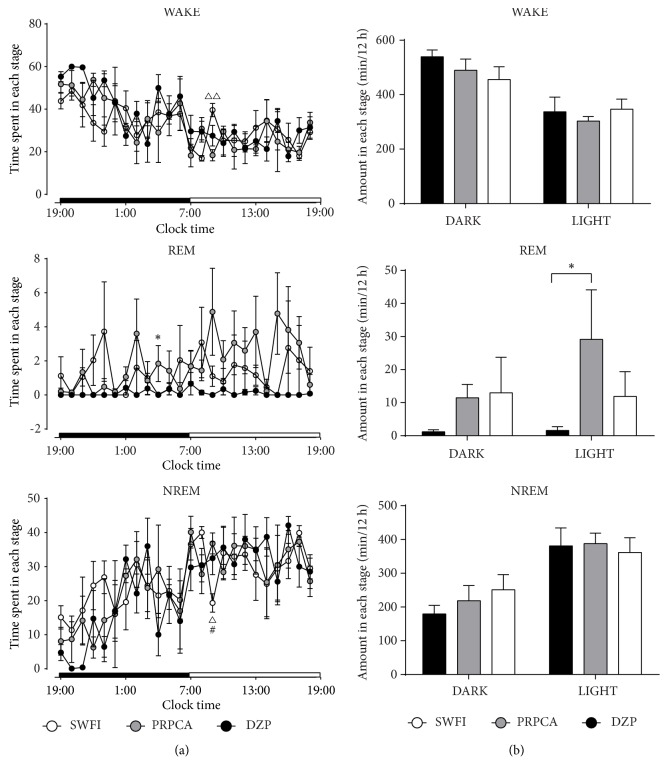
*Sleep-stage distributions produced by SWFI, PRPCA, and DZP*. (a) Time course of changes in wakefulness, REM sleep, and NREM sleep. Each circle represents the mean hourly amount of time spent in each stage. The horizontal open and filled bars on the X-axes indicate the 12-h light and 12-h dark periods, respectively. (b) Total time spent in wakefulness, REM sleep, and NREM sleep during the 12-h light and 12-h dark periods. White, gray, and black circles/bars represent the profiles for the SWFI, PRPCA, and DZP groups, respectively. Data are expressed as the mean ± SEM; ^#^P<0.05: PRPCA vs. SWFI; *∗*P<0.05: PRPCA vs. DZP; ^∆∆^P<0.01 and ^∆^P<0.05: SWFI vs. DZP as assessed using ANOVA followed by the LSD test. SWFI: sterile water for injection; PRPCA:* Pinelliae Rhizoma Praeparatum Cum Alumine*; DZP: diazepam; REM: rapid eye movement; NREM: nonrapid eye movement; ANOVA: analysis of variance: LSD: least significant difference.

Based on amplitude and frequency results from the EEG/EMG analyses, sleep–wake stages were divided into wakefulness, NREM sleep, and REM sleep [21]. As shown in Figure 2(a), PRPCA decreased the hourly wakefulness time by 46.30% at 9:00 AM (∆P<0.05) and increased the hourly NREM sleep time by 1.90-fold at 9:00 AM (∆P<0.05), when compared with SWFI. PRPCA also increased the hourly REM sleep time by 92-fold at 4:00 AM (*∗*P<0.05), relative to DZP. DZP increased the hourly NREM sleep time by 1.68-fold at 9:00AM (^#^P<0.05), when compared with SWFI. Increases in NREM sleep coincided with decreases in wakefulness.

The total amount of time spent in wakefulness, NREM, and REM sleep is summarized in Figure 2(b). PRPCA increased REM sleep by 18-fold during the light phase (*∗*P<0.05), relative to DZP. No other significant differences were observed.

### 3.3. Changes in the Number of Bouts Induced by PRPCA

**Figure 3:**
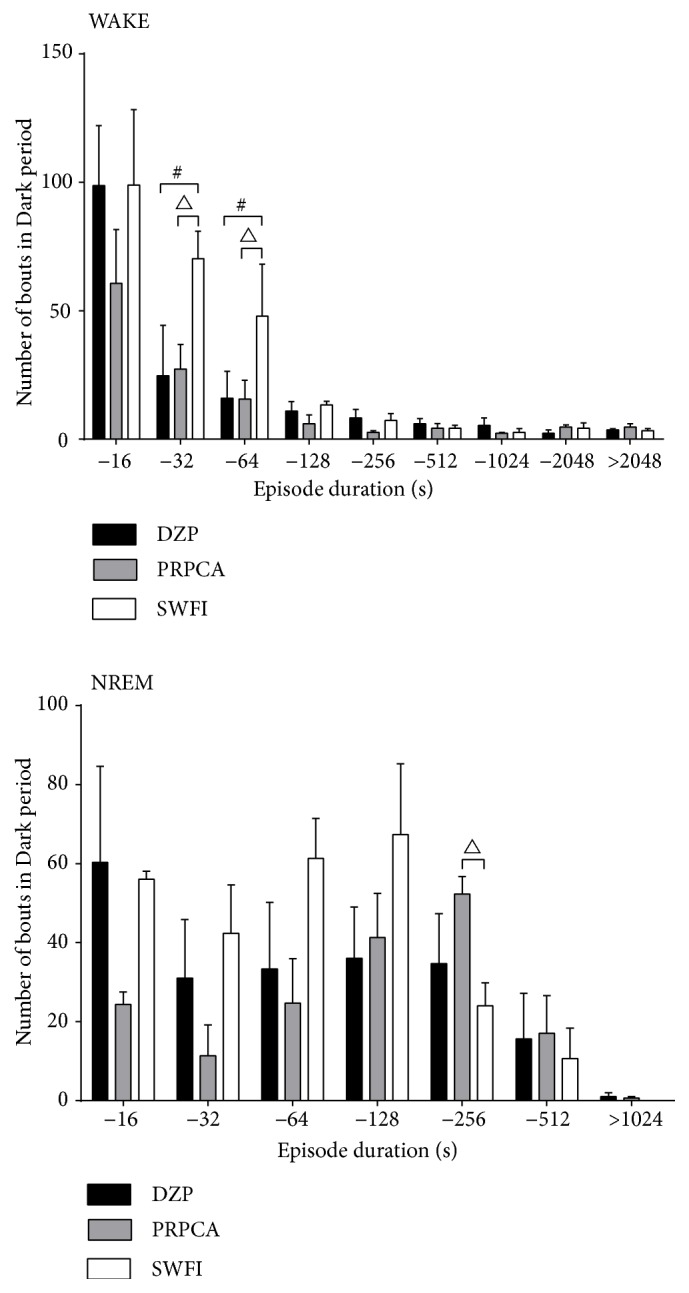
*Bouts of wakefulness and NREM sleep during the dark period*. White, gray, and black bars show the profiles for the SWFI, PRPCA, and DZP groups, respectively. The negative time values on the X-axis represent the following periods, respectively: 0–16 s, 16–32 s, 32–64 s, 64–128 s, 128–256 s, 256–512 s, 512–1024 s, and 1024–2048 s. Data are expressed as the mean ± SEM; ^#^P<0.05: PRPCA vs. SWFI; ^∆^P<0.05 SWFI vs. DZP as assessed using ANOVA followed by the LSD test. NREM: nonrapid eye movement; SWFI: sterile water for injection; PRPCA:* Pinelliae Rhizoma Praeparatum Cum Alumine*; DZP: diazepam; ANOVA: analysis of variance; LSD: least significant difference.

To better understand the sleep–wake profile induced by PRPCA, we examined the distributions of bouts of wakefulness, NREM, and REM sleep as a function of the duration of each bout or episode (Figure 3). PRPCA decreased the number of bouts of wakefulness with durations of 16–32s and 32–64s by 38.91% and 32.68% (^∆^P<0.05, ^∆^P<0.05), respectively, and increased the number of bouts of NREM sleep with durations of 128–256s by 2.18-fold (^∆^P<0.05) during the dark phase, relative to SWFI. In addition, DZP decreased the number of bouts of wakefulness with durations of 16–32s and 32–64s by 35.09% and 33.33% (^∆^P<0.05, ^∆^P<0.05), respectively. However, we observed no significant changes in the transition between different sleep stages or the number of episodes for different sleep stages.

### 3.4. Characteristics of the Compounds in PRPCA

**Figure 4:**
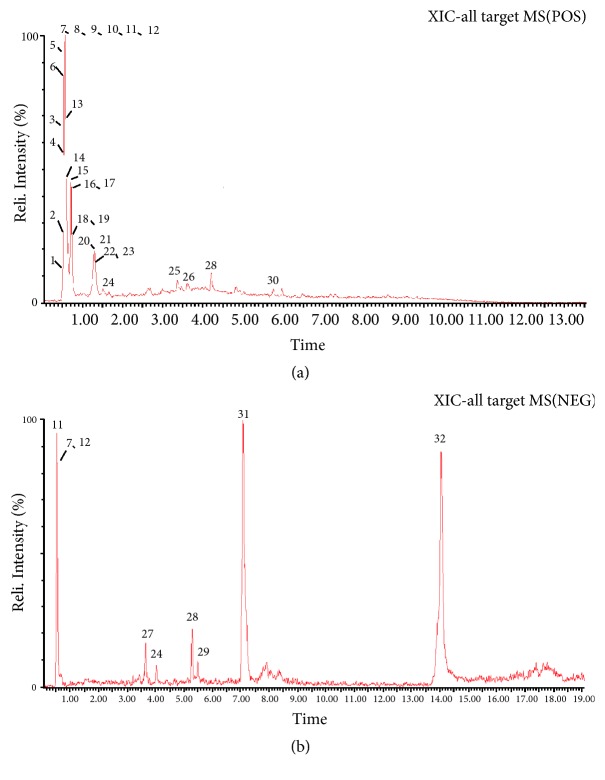
*Extracted ion chromatogram of compounds in PRPCA identified via UPLC/Q-TOF-ESI/MS*. (a) Extracted ion chromatogram in positive mode (ESI+). (b) Extracted ion chromatogram in negative mode (ESI–). PRPCA:* Pinelliae Rhizoma Praeparatum Cum Alumine*; UPLC: ultra-performance liquid chromatography; Q-TOF: quadrupole time-of-flight; ESI: electrospray ionization; MS: mass spectrometry.

The identities of all detected peaks in PRPCA were determined via UPLC/Q-TOF-MS. A total of 32 compounds were identified and categorized into 19 alkaloids, three volatile oils, two fatty acids, two phenylpropanoids, and others. The representative LC–MS chromatograms are shown in Figure 4.  Table 4 lists the characteristics of the 32 identified compounds.

## 4. Discussion

In the present study, we investigated the sedative effects of different concentrations of PRPCA, observing that lower concentrations of PRPCA were more effective at inducing sedation than higher concentrations, when compared with SWFI. The relatively worse sedative effects of PRPCA 2 and PRPCA 3 may be due to an inability to absorb and utilize such high concentrations of the drug. Thus, our results highlight the need for further studies regarding the most appropriate concentration of PRPCA for treatment, as even lower concentrations may be effective.

In addition, we observed that successive PRPCA administration was associated with reduced locomotor activity in mice, suggesting a cumulative effect of PRPCA treatment. Furthermore, locomotor activity counts among the different groups at the same time differed beginning on day 8, suggesting that PRPCA should be successively administered for more than 8 days to achieve a better curative effect. Unfortunately, we were unable to determine an unequivocal time course for treatment because the experiment was performed for a limited number of days, necessitating further studies with longer experimental periods.

We then investigated the hypnotic effects and mechanisms underlying the efficacy of PRPCA via sleep and vigilance state analyses. Our results indicated that PRPCA reduced hourly wakefulness time and increased hourly NREM sleep time at 9:00 AM (Figure 2(a)). In addition, PRPCA treatment increased the amount of 12-h REM sleep time during the light phase. Such results indicate that the hypnotic effects of PRPCA occurred in accordance with the physiological sleep–wake rhythm of mice. Further analysis revealed that PRPCA treatment reduced the number of bouts of wakefulness with shorter durations (16–32s, 32–64s) and increased the number of bouts of NREM sleep with longer durations (128–256s), suggesting that PRPCA can improve sleep fragmentation and sleep quality.

In order to provide insight into the extraction of drug monomers and inform the preparation of safer, more convenient drugs, we utilized chromatography and MS to detect the main chemical components of PRPCA. We identified 32 compounds in PRPCA, including 19 alkaloids, three volatile oils, two phenylpropanoids, two fatty acids, one cerebroside, one anthraquinone, and four others. When considering relative abundance, alkaloids accounted for 32.74%, fatty acids accounted for 49.73%, and volatile oils accounted for 3.09% of PRPCA components. These three types of components accounted for 85.56% of the total ingredients identified. One previous study reported that PR is rich in alkaloids, volatile oils, fatty acids, and other ingredients, which can reduce the activity of some enzymes in tissue cells, thereby inhibiting the central nervous system and playing a role in sedation and hypnosis [22]. Such results provide a range of options for subsequent studies regarding drug monomers.

However, the present study possesses some limitations of note. First, further studies involving longer experimental periods are required to determine the most appropriate concentration and unequivocal time course for treatment. Second, although we demonstrated that PRPCA can promote sleep by reducing wakefulness and increasing sleep time, the underlying physiological principles remain to be elucidated. Third, the roles of the 32 identified PRPCA components in promoting sleep must be investigated in future studies.

## 5. Conclusion

Our findings demonstrated that PRPCA exerts cumulative sedative and hypnotic effects. PRPCA improved sleep quality by increasing REM sleep and reducing sleep fragmentation in accordance with the physiological sleep–wake rhythm of mice, suggesting that PRPCA can be used in the clinical treatment of insomnia. Although alkaloids and fatty acids appear to play a major role, the mechanisms underlying the sedative and hypnotic effects of PRPCA remain to be fully elucidated.

## Figures and Tables

**Table 1:** Impact of time and group on locomotor activity in mice.

	F	P
Time	2.133	0.088
Group	4.267	≤0.001
Time*∗*Group	6.242	0.004

**Table 2:** Comparison among groups at the same time point.

	d8	d9	d10	d11	d12	d13	d14
F	8.17	6.937	6.733	7.416	7.526	18.346	13.872
P	0.001^*∗∗*^	0.002^*∗∗*^	0.003^*∗∗*^	0.002^*∗∗*^	0.002^*∗∗*^	0^*∗∗*^	0^*∗∗*^

**Table 3:** Average locomotor activity in each group (count/24h).

	DZP	PRPCA 1	PRPCA 2	PRPCA 3	SWFI
$\overset{-}{X}$	50226.04	37239.75	48008.89	47746.07	62956.95
S	11057.37	9772.69	9638.59	9940.67	13226.20
DZP		0.025^*∗∗*^	0.676	0.640	0.027^*∗∗*^
SWFI		≤0.001^∆∆^	0.012^∆∆^	0.010^∆∆^	

^*∗∗*^P value vs. DZP, ^∆∆^ P value vs. SWFI. DZP: diazepam; PRPCA: *Pinelliae Rhizoma Praeparatum Cum Alumine*; SWFI: sterile water for injection.

**Table 4:** Chemical components of PRPCA.

No.	Rt(min)	[M+H]^+^/[M-H]^−^(m/z)^1^	Relative abundance^2^ (%)	Formula	Identification	Component type
1	0.49	175.1055/	0.846276	C_6_H_14_N_4_O_2_	L-arginine	Alkaloid
2	0.51	134.0433/	1.543210	C_4_H_7_NO_4_	Aspartic acid	Alkaloid
3	0.53	173.2322/	20.549580	C_9_H_16_O_3_	9-Oxo-nonanoic acid	Fatty acid
4	0.53	104.0692/	1.931501	C_4_H_9_NO_2_	r-Aminobutyric acid	Alkaloid
5	0.55	90.0647/	1.324170	C_3_H_7_NO_2_	Alanine	Alkaloid
6	0.55	116.0694/	0.667065	C_5_H_9_NO_2_	Proline	Alkaloid
7	0.56/ 0.56	343.1393/ 341.1121	17.502990/ 9.261898	C_16_H_22_O_8_	Coniferin	Phenylpropanoid
8	0.56	127.0395/	0.696933	C_6_H_6_O_3_	5-Hydroxymethylfurfural	Alkaloid
9	0.56	127.0395/	0.527680	C_5_H_6_N_2_O_2_	5-Methyl uracil	Alkaloid
10	0.56	181.0703/	1.971326	C_9_H_8_O_4_	Caffeic acid	Other
11	0.56/ 0.55	145.0491/ 143.0344	2.031063 /1.082930	C_6_H_8_O_4_	1,6:3,4-Dianhydro-*β*-D-allosep	Other
12	0.56/ 0.56	343.122/ 341.122	17.562720 /9.233400	C_12_H_22_O_11_	Sucrose	Phenylpropanoid
13	0.57	148.0641/	0.975707	C_5_H_9_NO_4_	Glutamic acid	Alkaloid
14	0.60	118.0849/	2.538829	C_5_H_11_NO_2_	Valine	Alkaloid
15	0.68	137.0473/	1.513341	C_5_H_4_N_4_O	6- purine	Alkaloid
16	0.69	182.0808/	1.184787	C_9_H_11_NO_3_	Tyrosine	Alkaloid
17	0.69	150.0623/	0.796495	C_5_H_11_O_2_NS	Methionine	Alkaloid
18	0.71	132.101/	2.489048	C_6_H_13_NO_2_	Isoleucine	Alkaloid
19	0.71	136.0645/	3.803266	C_5_H_5_N_5_	Pedatisectine B	Alkaloid
20	1.28	166.0844/	6.800080	C_9_H_11_NO_2_	Phenylalanine	Alkaloid
21	1.29	120.0781/	1.324174	C_4_H_9_NO_3_	Threonine	Alkaloid
22	1.30	167.0919/	0.527678	C_10_H_14_O_2_	5-Amyl-2-pyrone	Volatile oil
23	1.30	268.1042/	2.220231	C_10_H_13_N_5_O_4_	Adenosine	Alkaloid
24	1.50/ 4.06	263.1381/ 261.1375	1.702509/ 1.738387	C_14_H_18_N_2_O_3_	Cyclo-(Val-L-Tyr)	Alkaloid
25	3.35	696.538/	1.403823	C_40_H_73_NO_8_	1-O-glucosyl-N-2′-palmitoyl-4,8-sphingodienine	Cerebroside
26	3.59	97.0749/	1.652728	C_6_H_8_O	2-Ethenyl butenal	Volatile oil
27	/3.68	/167.0421	1.766885	C_8_H_8_O_4_	Vanillic acid	Other
28	4.18/ 5.32	255.0653/ 253.0549	2.359618 /4.502707	C_15_H_10_O_4_	Chrysophanol	Anthraquinone
29	5.51	/191.0798	0.911941	C_15_H_12_	Methyl phenanthrene	Volatile oil
30	5.72	269.0709/	1.553166	C_10_H_12_N_4_O_5_	Inosine	Alkaloid
31	/7.11	/329.2371	29.182100	C_18_H_34_O_5_	Pinellic acid	Fatty acid
32	/14.05	/293.1834	40.609860	C_17_H_26_O_4_	Gingerol	Other

Note 1: Data to the left of “/” represent the m/z of [M+H]. Data to the right of “/” represent the m/z of [M-H]. Note 2: Relative abundance values were obtained via normalization.

## Data Availability

The data used to support the findings of this study are available from the corresponding author upon request.
